# Role of Heterotypic Neutrophil-in-Tumor Structure in the Prognosis of Patients With Buccal Mucosa Squamous Cell Carcinoma

**DOI:** 10.3389/fonc.2020.541878

**Published:** 2020-10-15

**Authors:** Jie Fan, Qigen Fang, Yang Yang, Meng Cui, Ming Zhao, Jinxing Qi, Ruihua Luo, Wei Du, Shanting Liu, Qiang Sun

**Affiliations:** ^1^ Department of Head Neck and Thyroid Surgery, Affiliated Cancer Hospital of Zhengzhou University, Henan Cancer Hospital, Zhengzhou, China; ^2^ Laboratory of Cell Engineering, Institute of Biotechnology, Beijing, China; ^3^ Department of Nephrology, The First Affiliated Hospital of Zhengzhou University, Zhengzhou, China; ^4^ Department of Anatomy, Zhengzhou University, Zhengzhou, China

**Keywords:** cell-in-cell, frequency of heterotypic neutrophil-in-tumor structure, buccal mucosa squamous cell carcinoma, prognosis, recurrence-free survival, disease-specific survival

## Abstract

**Objective:**

To analyze the role of frequency of heterotypic neutrophil-in-tumor structure (FNiT) in the prognosis of patients with buccal mucosa squamous cell carcinoma (BMSCC).

**Methods:**

*In vitro*, we cocultured BMSCC cell line-H157 with neutrophils to form heterotypic neutrophil-in-tumor structures, which were then subject to fluorescence staining. Clinically, 145 patients were retrospectively enrolled. Associations between FNiT and clinicopathological variables including age, sex, smoking history, drinking history, betel nut chewing, tumor stage, node stage, metastasis, disease stage, lymphovascular invasion, extranodal extension, perineural invasion, and tumor grade were analyzed by chi-square test, and the main endpoints of interest were recurrence-free survival (RFS) and disease-specific survival (DSS) which were analyzed by the Kaplan-Meier method and Cox model.

**Results:**

Fluorescent staining results of typical heterotypic neutrophil-in-tumor structure showed that well-differentiated H157 cells had a stronger ability to internalize more neutrophils than poorly-differentiated H157 cells, with the latter often internalizing only one neutrophil or nothing. The mean FNiT was 4.2‰, with a range from 2.3‰ to 7.8‰. A total of 80 patients relapsed and 84 patients died of the disease. The 5-year RFS and DSS rate was 42% and 42%, respectively. Patients with an FNiT≥4.2‰ had a significantly higher risk for locoregional recurrence and cancer-caused death than those with an FNiT<4.2‰ (p=0.001 and p<0.001, respectively). The FNiT alone was independently significant in predicting poor RFS, and the FNiT along with tumor grade was an independent predictor for DSS.

**Conclusion:**

The FNiT as a novel predictor is significantly negatively associated with both the RFS and DSS of patients with BMSCC.

## Introduction

Cell-in-cell (CIC) is an evolutionarily conserved cytobiological phenomenon (1, 2), which was first reported 150 years ago by a German scholar (3). It refers to the presence of one or more living cells within another living one and has ever since been found in varieties of tumors tissues, such as breast carcinoma (4, 5), pancreatic ductal adenocarcinoma (6), and head and neck squamous cell carcinoma (HNSCC) (7) and the like. CIC has two typical forms: one is homotypic CIC, and the other is heterotypic CIC, with the latter often occurring between tumor cells and immune cells that include neutrophils (8). Neutrophils are the most well-known marker and promoter of inflammation (9). Recent findings have indicated that inflammation, metabolic response, and immune response are the three main components of tumor microenvironment, which are of significance in cancer pathogenesis and progression by interacting with tumor cells (9–13). Several chemokines, cytokines and angiogenic factors produced by neutrophils may result in inflammatory cell recruitment and activation that crucially impact the tumor microenvironment, which could facilitate tumor cell proliferation, microvascular regeneration and tumor progression (9, 14, 15). The formation of CIC structure in tumors is a functional result of active intercellular interactions within heterogeneous tumor microenvironments, which is driven by a set of core molecular elements (16, 17) that are regulated by multiple factors, such as cholesterol and IL-8 (18–21).

Neutrophil-in-tumor (NiT) structures, previously found in HNSCC, are typical heterotypic CIC structures (hCIC), which refer to the presence of living neutrophils inside living tumor cells (7, 22, 23). The FNiT is defined as the **f**requency of heterotypic NiT structures formation and is calculated by the total number of NiT structures divided by the total number of tumor cells, reflecting the severity of tumor-infiltrating neutrophils in the tumor microenvironment based on our previous studies. Some clinical researches with limited cases had preliminarily found that NiT structures were associated with poor prognosis in patients with HNSCC (7, 23). However, the prognostic role of the FNiT in patients with BMSCC remained unclear.

## Materials and Methods

### In Vitro Study

#### Cells and Culture Conditions

H157 cells were maintained in Dulbecco’s modified Eagle’s medium supplemented with 10% fetal bovine serum (PAN-Biotech). Approximately 1 × 10^5^ H157 cells were adherently cultured in 12-well plates. Neutrophils were maintained in RPMI-1640 supplemented with 10% fetal bovine serum (PAN-Biotech). Approximately 1 × 10^6^ neutrophils were cultured in suspension in a 10-cm dish.

#### Heterotypic CIC Formation Assay

Briefly, approximately 1 × 10^5^ H157 cells were adherently cultured in 12-well plates for 8 h. Then, neutrophils and H157 cells were cocultured for 8 h. Cytospins were then made by centrifugation at 800 rpm for 4 min. Then, the cells were fixed and underwent both Phalloidin-568 and Hoechst staining to quantify hCIC structures. The presence of internalized cells wrapped by one outer cell was considered as one hCIC structure.

#### Fluorescence Staining

First, neutrophils were stained with CellTracker Green (Invitrogen). Second, cytospins were fixed in 4% paraformaldehyde and then proceeded to both routine Phalloidin-568 (Life) and Hoechst (Thermo) staining for 30 min before being mounted with Prolong Gold antifade reagent (Invitrogen). Confocal images were captured and processed by the Ultraview Vox confocal system (Perkin Elmer) on Nikon Ti-E microscope.

### Retrospective Case Series Study

#### Patients and Methods

The Institutional Research Committee of Henan Cancer Hospital approved our study. All patients participating in the study signed an informed consent agreement for medical research before initial treatment, and all methods were performed in accordance with relevant guidelines and regulations.

From March 2009 to October 2018, we conducted a retrospective study on 145 patients (≥29 years old) with previously untreated history undergoing radical resection of BMSCC (RRB). Of the enrolled patients, no one had synchronous head and neck carcinoma, immunological disorders, or previous chemotherapy and/or radiation of the head and neck area. Data regarding age, sex, smoker, drinker, betel nut chewing, FNiT, tumor stage, node stage, metastasis, disease stage, lymphovascular invasion, extranodal extension, perineural invasion, tumor grade, postoperative pathological report, operation record, adjuvant treatment, and follow-up information were extracted and analyzed. All pathologic sections made from primary tumor were re-evaluated *via* immunohistochemistry. In our cancer center, preoperative ultrasound and CT or MRI were routinely performed. The disease stage was defined based on the AJCC 8^th^ edition staging system, and the tumor grade was categorized according to the 2017 classification of the WHO. In addition, frozen sectioning of the primary tumor was routinely performed; if the pathology was malignant, a RRB was performed.

The FNiT was observed and calculated in the pathologic sections of BMSCC. The cutoff value calculated from the ROC curve, mean, tertile, or median in previous studies varied. Thus, the standard cutoff value remains unknown. In the current study, the cutoff value was defined as the mean value of the FNiT according to relevant published reports.

The chi-squared test was used to assess the association between the FNiT and the clinicopathological variables. The Kaplan-Meier method was used to calculate the RFS and DSS rates. The Cox proportional hazards method was used to determine the independent risk factors for RFS and DSS. All statistical analyses were conducted with the help of SPSS version 20 (IBM Corporation, Armonk, NY, USA). A P<0.05 was considered significant.

#### Immunohistochemistry Staining

Prepared pathological sections were blocked with 5% (w/v) BSA for 1 h at room temperature followed by incubation with the primary antibodies against E-cadherin overnight at 4°C, HRP-conjugated secondary antibodies (1:2000) were applied for 1 h at room temperature before developed by DAB reagent.

## Results

### In Vitro Study

**Figure 1:**
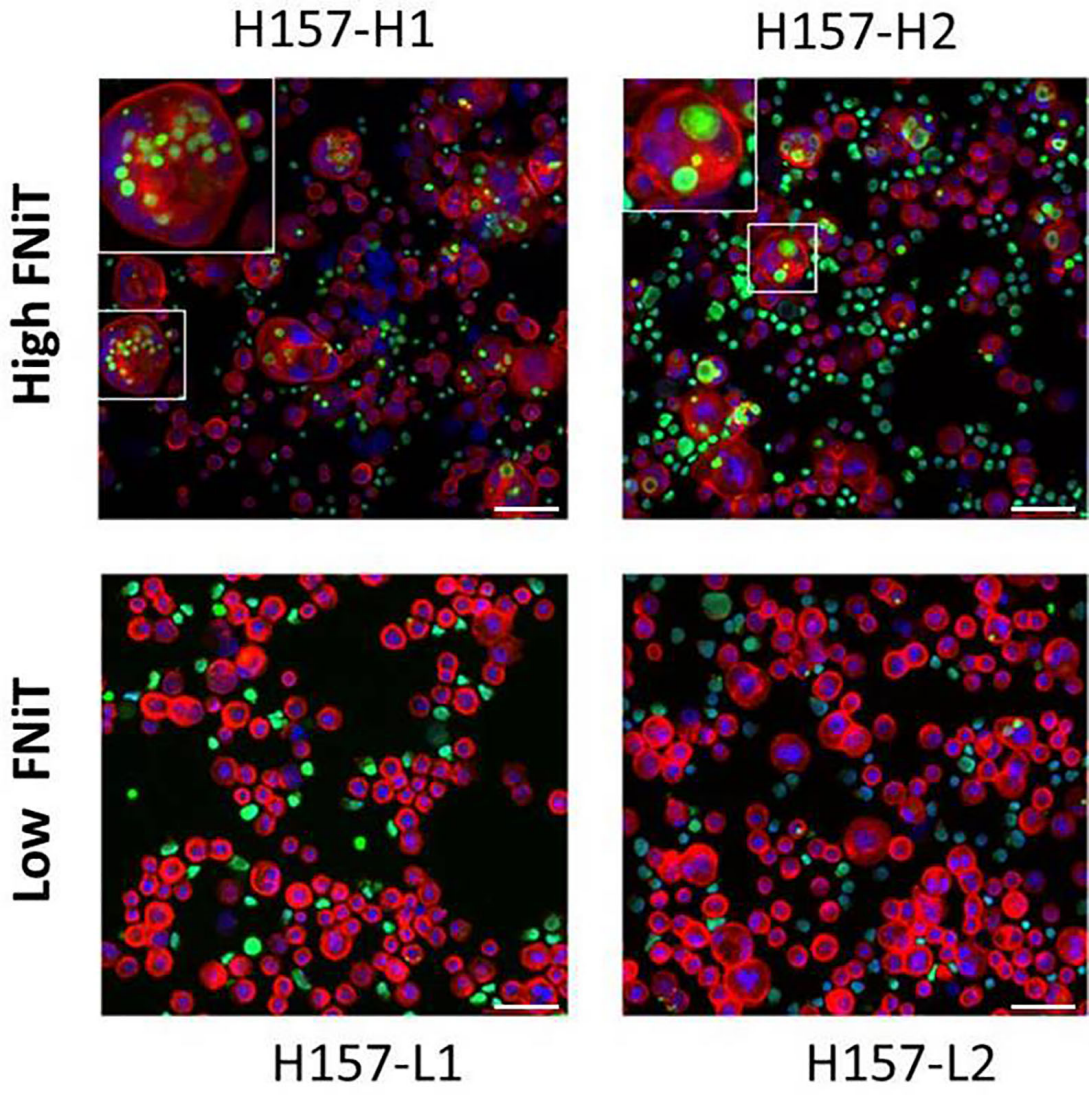
Fluorescent staining result of typical heterotypic NiT structure formed between H157 cells and neutrophils. H157-H1 and H157-H2 are well-differentiated BMSCC cell lines with high FNiT, and H157-L1 and H157-L2 are poorly differentiated BMSCC cell lines with low FNiT. Cells marked in red and green are H157 cells and neutrophils, respectively. The regions marked in blue are the nuclei of H157 cells and neutrophils. Scale bar of all: 100 mm.

In vitro, we cocultured BMSCC cell line-H157 with neutrophils to form NiT structures. Fluorescent staining results of typical heterotypic NiT structures are shown in **Figure 1**. H157-L1 and H157-L2 were two subpopulations of poorly differentiated BMSCC cell lines, while H157-H1 and H157-H2 were two subpopulations of well-differentiated BMSCC cell lines. Cells marked in red and green were H157 and neutrophils, respectively. The region marked in blue was the nuclei of H157 and neutrophils. We discovered that well-differentiated H157-H1 and H157-H2 had stronger ability to internalize more neutrophils than poorly differentiated H157-L1 and H157-L2, with the latter often internalizing only one neutrophil or nothing.

### Retrospective Case Series Study

Clinically, in total, 145 patients (68 females and 77 males) were enrolled with a mean age of 56.4 (range: 29–87) years. An FNiT≥4.2‰ was detected in 78 (54%) patients, while an FNiT<4.2‰ was detected in 67 (46%) patients. A history of smoking was found in 81 (56%) patients. A history of drinking was noted in 45 (31%) patients. Betel nut chewing was prevalent in 15 (10%) patients. Tumor stage was distributed as follows: T1 in 43 (30%) patients, T2 in 22 (15%) patients, T3 in 55 (38%) patients, and T4 in 25 (17%) patients. Distant metastasis was noted in 11 (8%) patients. Lymphovascular invasion was noted in 10 (7%) patients. Extranodal extension was found in 21 (14.5%) patients. Perineural invasion was noted in 8 (5.5%) patients. Tumor grade was distributed as follows: low grade in 10 (7%) patients, median grade in 26 (18%) patients, high grade in 109 (75%) patients (**Table 1**). A negative margin was achieved in 145 (100%) patients. The mean FNiT was 4.2‰, with a range from 2.3‰ to 7.8‰.

**Table 1:** General clinicopathological information of enrolled patients.

Variables	Number(%)
Age(years)	
<60	67(46%)
≥60	78(54%)
Sex	
Male	77(53%)
Female	68(47%)
FNiT	
Low	67(46%)
High	78(54%)
Smoker	
Y	81(56%)
N	64(44%)
Drinker	
Y	45(31%)
N	100(69%)
Betel nut chewing	
Positive	15(10%)
Negative	130(90%)
Tumor stage	
T1+T2	65(45%)
T3+T4	80(55%)
Node stage	
N0	100(69%)
N+	45(31%)
Metastasis	
Positive	11(8%)
Negative	134(92%)
Disease stage	
I+II	59(41%)
III+IV	86(59%)
Lymphovascular invasion	
Positive	10(7%)
Negative	135(93%)
Locoregional recurrence	
Y	80(55%)
N	65(45%)
Extranodal extension	
Y	21(14.5%)
N	124(85.5%)
Perineural invasion	
Y	8(5.5%)
N	137(94.5%)
Tumor Grade	
Low	10(7%)
Median	26(18%)
High	109(75%)

In total, all the patients underwent RRB and neck dissection whatever the clinical node stage. Pathologically negative and positive neck disease was reported in 100 (69%) and 45 (31%) patients, respectively (**Table 1**). A total of 110 patients received adjuvant radiotherapy, and adjuvant chemotherapy was performed in 29 patients.

**Figure 2:**
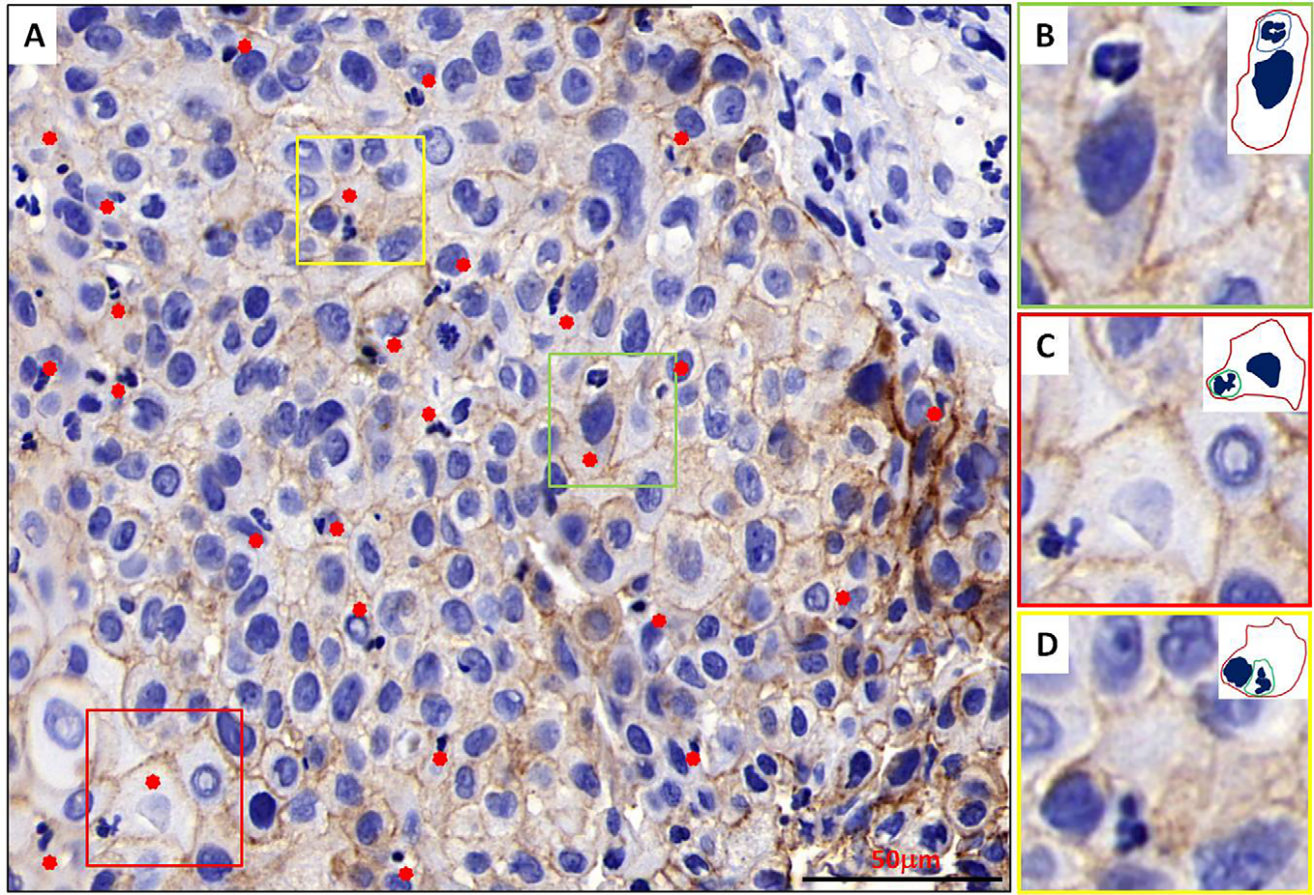
Typical NiT structures formation in BMSCC tissue. **(A)** Representative image for E-cadherin staining in BMSCC pathologic tissue with extensive neutrophils infiltration. Typical NiT structures are indicated with red asterisks. Scale bar: 50 mm. **(B–D)** Zoomed in images for boxed NiT structures in **(A)**. Each of them is one typical NiT structure. Inserted pictures of each image are schematic cartoons for the indicated NiT structures. FNiT=t/T. (t: the total number of NiT structures. T: the total number of the tumor cells.)

When re-evaluating all pathologic sections made from primary tumors *via* immunohistochemistry, we discovered the existence of typical NiT structures formation in BMSCC tissue (**Figure 2**). Representative image for E-cadherin staining in BMSCC pathologic tissue showed that tumor tissue was infiltrated with extensive neutrophils and substantial NiT structures were formed by tumor cells internalizing neutrophils (**Figure 2A**). Typical NiT structures were indicated with red asterisks, of which three boxed NiTs in **Figure 2A** were zoomed in as shown in **Figures 2B–D**. Each of them was one typical NiT structure. The inserted picture of each image was a schematic cartoon for the indicated NiT structure. We calculated the FNiT value of each pathologic section according to the formula: FNiT=**t**/**T** (**t**: the total number of NiT structures; **T**: the total number of the tumor cells).

**Figure 3:**
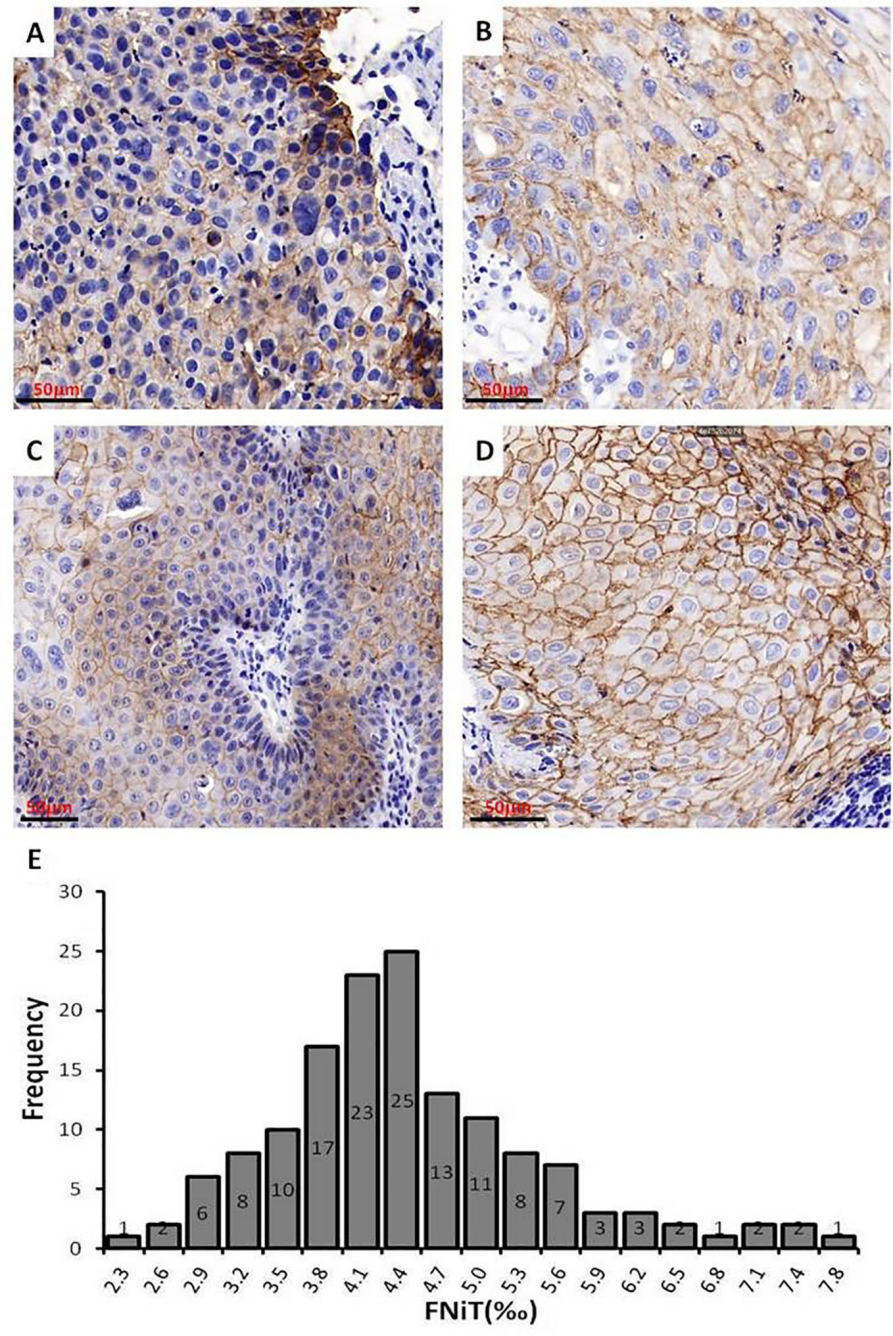
Images of BMSCC tissue with different levels of FNiT and FNiT distribution in enrolled patients. **(A, B)** Representative images of pathologic tissues with high FNiT in two patients with BMSCC. Scale bar: 50 mm. **(C, D)** Representative images of pathologic tissues with low FNiT in two patients with BMSCC. Scale bar: 50 mm. **(E)** Histogram plot for FNiT distribution in enrolled patients. The cutoff value of the FNiT was calculated by the mean value of the FNiT in enrolled patients.

We exhibited the images of BMSCC tissue with different levels of FNiT and FNiT distribution in enrolled patients in **Figure 3**. In **Figures 3A, B**, two representative pathologic tissues from two patients with BMSCC were shown with high FNiT (7.8‰ and 6.6‰, respectively). In **Figures 3C, D**, two representative pathologic tissues from two patients with BMSCC were shown with low FNiT (2.3‰ and 2.5‰, respectively). Histogram plot for FNiT distribution in all enrolled patients was shown in **Figure 3E**. The cutoff value of FNiT calculated by the mean value of FNiT in all the enrolled patients was 4.2‰.

When analyzing the association between the FNiT and clinicopathological variables, it was noted that the FNiT was significantly associated with tumor grade (**Table 2**).

**Table 2:** Association between FNiT and clinicopathological characteristics.

Variables	FNiT		P-value
	Low (<4.2‰) n = 67	High (≥4.2‰) n = 78	
Age(years)			0.749
<60	30	37	
≥60	37	41	
Sex			0.298
Male	32	44	
Female	35	34	
Smoker			0.886
Y	37	44	
N	30	34	
Drinker			0.519
Y	19	26	
N	48	52	
Betel nut chewing			0.291
Positive	5	10	
Negative	62	68	
Tumor stage			0.510
T1+T2	32	33	
T3+T4	35	45	
Node stage			0.941
N0	46	54	
N+	21	24	
Metastasis			0.190
Positive	3	8	
Negative	64	70	
Disease stage			0.353
I+II	30	29	
III+IV	37	49	
Lymphovascular invasion			0.085
Positive	2	8	
Negative	65	70	
Extranodal extension			0.697
Y	9	12	
N	58	66	
Perineural invasion			0.296
Y	3	5	
N	65	72	
Tumor Grade			<0.001
Low	8	2	
Median	19	7	
High	40	69	

**Figure 4:**
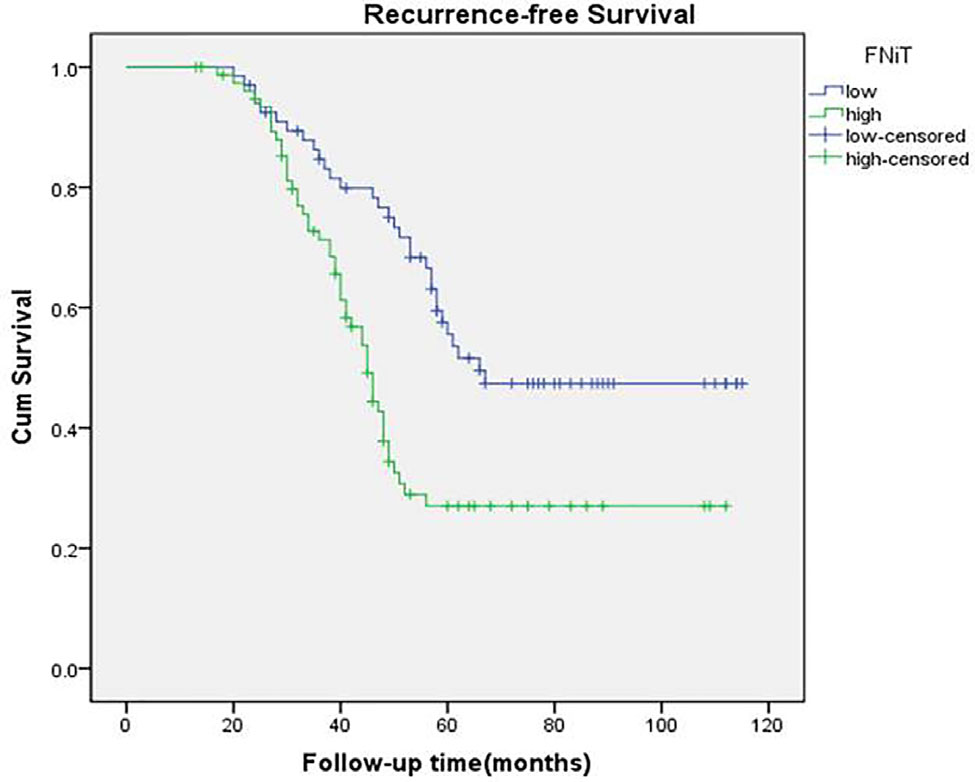
Recurrence-free survival function in patients with different FNiT (p = 0.018).

During our follow-up with a mean time of 52.4 (range: 13–115) months, a total of 80 (55%) patients relapsed locoregionally. Salvage surgery was performed successfully in 65 patients by local resection of BMSCC or radical neck dissection. The 5-year RFS rate was 42%, with 54.2 months of median survival time of RFS. When evaluating the predictors for RFS in the univariate analysis, betel nut chewing, FNiT, tumor stage, metastasis, disease stage, and tumor grade were significantly associated with locoregional recurrence (LRR). Furthermore, the FNiT was confirmed as the only independent predictor for RFS in the Cox model (**Table 3**). In patients with an FNiT<4.2‰, the 5-year RFS rate was 56%, and in patients with an FNiT≥4.2‰, the 5-year RFS was 27%; the difference was significant (p=0.018, **Figure 4**).

**Table 3:** Univariate and multivariate analyses of predictors for recurrence-free survival in patients with BMSCC.

Variables	Univariate	Cox model	
	P-value	P-value	OR(95% CI)
Age,years(<60 vs ≥60)	0.591		
Sex(male vs female)	0.220		
Smoker (Y vs N)	0.681		
Drinker (Y vs N)	0.580		
Betel nut chewing(Y vs N)	<0.001	0.180	2.046(0.718–5.831)
FNiT(Low vs High)	0.001	0.018	1.803(1.106–2.940)
Tumor stage(T1+T2 vs T3+T4)	0.016	0.953	1.036(0.327–3.276)
Node stage(N0 vs N+)	0.342		
Metastasis(Y vs N)	<0.001	0.744	1.252 (0.325–4.821)
Disease stage(I+II vs III+IV)	0.001	0.313	1.907(0.545–6.671)
Lymphovascular invasion(Y vs N)	0.634		
Extranodal extension(Y vs N)	0.268		
Perineural invasion(Y vs N)	0.521		
Tumor Grade	0.042	0.176	1.365(0.870–2.143)
Low			
Median			
High			

**Figure 5:**
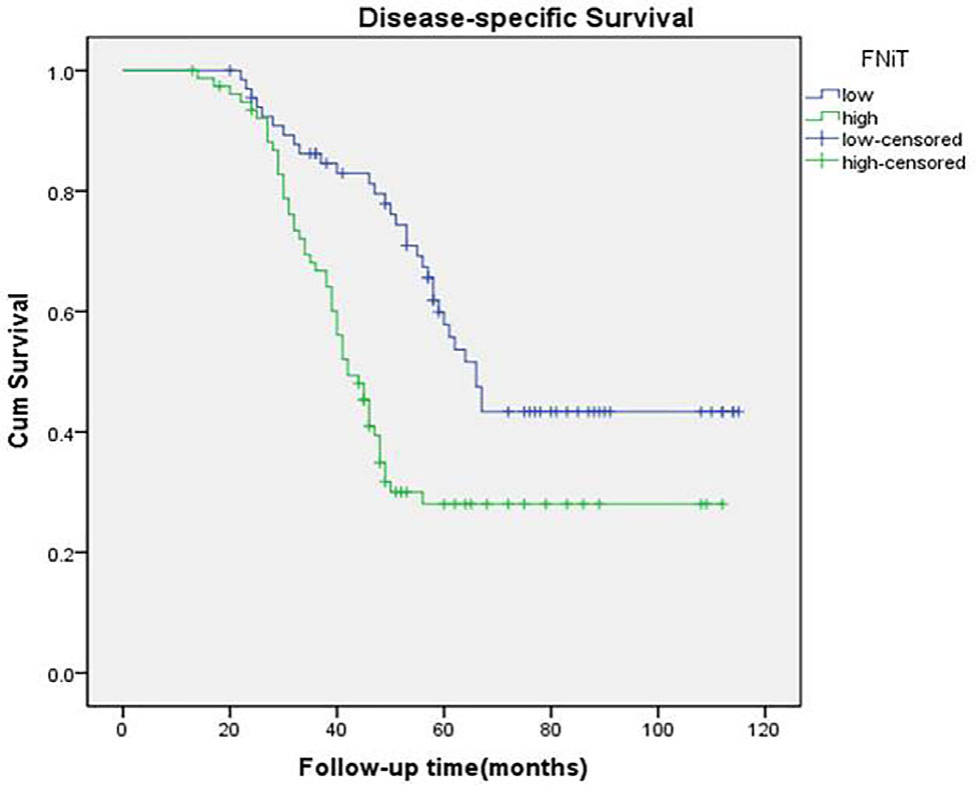
Disease-specific survival function in patients with different FNiT (p = 0.034).

**Figure 6:**
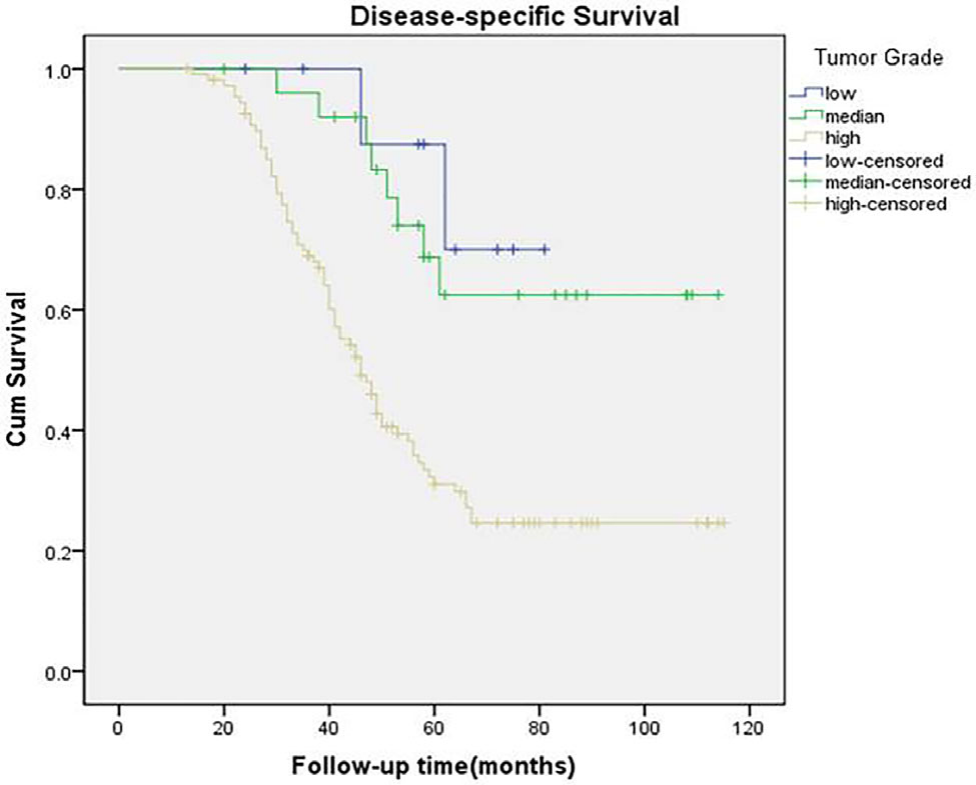
Disease-specific survival function in patients with different levels of tumor grade (p = 0.003).

A total of 84 patients died of the disease. The 5-year DSS rate was 42% with 54.0 months of median survival time of DSS. In the univariate analysis, betel nut chewing, FNiT, tumor stage, metastasis, disease stage, lymphovascular invasion and tumor grade were significantly associated with terminal death. The Cox model was further utilized to identify that the FNiT and tumor grade were two independent factors predicting DSS in patient with BMSCC (**Table 4**). In patients with an FNiT<4.2‰, the 5-year DSS rate was 58%, and in patients with an FNiT≥4.2‰, the 5-year DSS rate was 28%; the difference was significant (p=0.034, **Figure 5**). Patients with high tumor grade tended to have a shorter DSS than those with low or median tumor grade (p=0.003, **Figure 6**).

**Table 4:** Univariate and multivariate analyses of predictors for disease-specific survival in patients with BMSCC.

Variables	Univariate	Cox model	
	P-value	P-value	OR(95% CI)
Age,years(<60 vs ≥60)	0.456		
Sex(male vs female)	0.558		
Smoker (Y vs N)	0.993		
Drinker (Y vs N)	0.701		
Betel nut chewing(Y vs N)	0.001	0.236	1.888(0.660–5.398)
FNiT(Low vs High)	<0.001	0.034	1.677(1.039–2.706)
Tumor stage(T1+T2 vs T3+T4)	0.001	0.941	0.954(0.275–3.305)
Node stage(N0 vs N+)	0.533		
Metastasis(Y vs N)	0.002	0.907	0.920 (0.228–3.709)
Disease stage(I+II vs III+IV)	<0.001	0.174	2.524(0.664–9.586)
Lymphovascular invasion(Y vs N)	0.038	0.068	2.122(0.946–4.757)
Extranodal extension(Y vs N)	0.467		
Perineural invasion(Y vs N)	0.573		
Tumor Grade	<0.001	0.003	2.371(1.337–4.203)
Low			
Median			
High			

## Discussion

Heterotypic CIC structures are generally formed by internalization of immune cells into tumor cells (24). Many tumor cells were confirmed to have the ability to internalize immune cells (25, 26), for example, HNSCC, melanoma, ductal carcinoma of the salivary gland, breast cancer, liver cancer and other tumor cells (8, 22, 27). The immune cells engulfed by tumor cells are diverse, including neutrophils (14, 15) NK cells (24, 28), T lymphocytes, and LAK cells, and neutrophils were recently mostly investigated. It has been reported that tumor progression and prognosis are associated with hCIC structure to some extent. Tetikkurt (7) and Sarode (23) described the inverse association between frequency of hCIC structure and prognosis of HNSCC, which indicates that hCIC plays an important role in predicting the progression of HNSCC.

Tumor cells internalize neutrophils to form heterotypic neutrophil-in-tumor structures, which have been discovered in HNSCC as a novel phenomenon (8, 29, 30). In our study, we cocultured BMSCC cell line-H157 with neutrophils *in vitro* to form NiT structures. We noted that H157-H1 and H157-H2 had higher ability to internalize more neutrophils than H157-L1 and H157-L2. The former cells were generally morphologically better differentiated than the latter ones. We noted that tumor grade was positively correlated with the FNiT in the association between the FNiT and clinicopathological characteristics. That is, the FNiT in well-differentiated tumor tissue is lower than that of poorly differentiated tumor tissue. This is not contradictory to the findings of the *in vitro* hCIC assays mentioned above. We supposed that the tumor tissues with high FNiT have more neutrophil infiltration than those with low FNiT, although the proportion of well-differentiated tumor cells in the low-FNiT tumor tissues surpassed that in the high-FNiT tumor tissues. We could further infer that the level of FNiT value was mainly attributed to the neutrophils infiltration within tumor tissues rather than the proportion of well-differentiated tumor cells, and the neutrophils infiltration within tumor tissues had a vital impact on the prognosis in patients with BMSCC.

To date, the role of the FNiT in the prognosis of patients with BMSCC has scarcely been investigated. A few papers have reported the existence of neutrophil-in-tumor structures in other tumors of HNSCC. Arya et al. (8) reported striking neutrophil-in-tumor cell cannibalism associated with a high grade, aggressive and metastatic duct carcinoma of the parotid gland. Magalhaes et al. (29) reviewed the role of neutrophils in the tumor microenvironment and as signaling modulators of oral squamous cell carcinoma (OSCC) and their possible role as biomarkers of OSCC prognosis and reported a pro-tumor role for NiT structure in OSCC. Tetikkurt et al. (7) presented a case related to significant neutrophilic emperipolesis in squamous cell carcinoma of the hard palate and maxilla and found that patients with high frequency of neutrophil-in-tumor structure were prone to relapse. Furthermore, neutrophil-in-tumor structures have been identified in other solid tumors such as microscopy evaluation of pleomorphic cell (giant cell) carcinomas of the lung, invasive micropapillary carcinomas of the ampulopancreaticobiliary region, gastric carcinomas, and lymphomas (31–33).

For BMSCC, alcohol consumption, tobacco use and betel quid chewing are well-known risk factors (34, 35). In the current study, we demonstrated that 31% of the patients had a history of alcohol consumption, and 56% had a history of tobacco product consumption, which is consistent with previous studies. However, the proportion of betel nut chewing was 10%, which was lower than the reported result. We attributed the regional differences and human species diversity to the abnormal phenotype, as people in Hunan Province of China tended to consume more betel nuts than the ones in other region. For prognosis of BMSCC, we discovered that betel nut chewing was significantly associated with RFS and DSS (p<0.001 and p=0.001, respectively). This was in accordance with previous studies.

The LRR rate of BMSCC was generally high, as published papers have already indicated. DeConde et al. (36) found that 21 (44%) out of 48 patients with BMSCC relapsed during postoperative follow-up. In our current study, the LRR rate was 55%, which was higher than the average value previously reported. First, we speculated that it was possibly due to the high proportion (75%) of high tumor grade of the enrolled patients. Secondly, regional lymph node metastasis occurred in 45 (31%) of the 145 total patients, which was relatively higher than previous results. Coppen et al. (37) reported that the prevalence of regional lymph node involvement was only 25%. Thirdly, although 110 (76%) out of 145 patients received postoperative radiotherapy, the majority of relapsed patients were continuously addicted to tobacco or alcohol.

We innovatively investigated the prognostic role of the FNiT in patients with BMSCC in the current study. We fortunately discovered that the FNiT as a novel predictor is significantly independently associated with RFS and DSS. However, the mechanism underlying the association between the FNiT and prognosis of BMSCC remains unclear. As we indicated above, the FNiT reflects the proportion of tumor-infiltrating neutrophils within the tumor microenvironment. Several chemokines, cytokines, and angiogenic factors produced by neutrophils may induce inflammatory cell recruitment and activation that have an impact on the tumor microenvironment, which could in turn facilitate tumor cell proliferation, microvascular regeneration and tumor progression (7, 9, 11, 13). In our study, patients with high FNiT tended to have both a shorter RFS and DSS than those with low FNiT, which may be better explained by the role of neutrophils in the tumor microenvironment mentioned above. Tetikkurt et al. (7) proposed that neutrophils may play a crucial role in cancer biology and may act as tumor promoters in tumor progression. Gregory et al. (38) declared that neutrophils may be vital biomarkers and targets for the administration and control of HNSCC.

In the analysis on the association between the FNiT and clinicopathological characteristics, we found that tumor stage was significantly associated with the FNiT (p<0.001). In the univariate analysis of the predictors for RFS in patients with BMSCC, betel nut chewing, FNiT, tumor stage, metastasis, disease stage, and tumor grade were proved to be associated with RFS; however, we unexpectedly discovered that only the FNiT was an independent predictor for RFS in Cox model. Particularly, our data indicated that tumor stage may be correlated with RFS dependent on FNiT (but not independently). In the univariate analysis of the predictors for DSS in patients with BMSCC, betel nut chewing, FNiT, tumor stage, metastasis, disease stage, lymphovascular invasion and tumor grade were related with DSS, but only the FNiT and tumor grade were independently associated with DSS. Other candidate predictors were not expected to be independently associated with RFS. Possible explanations may be that the proportions of the patients positive for betel nut chewing, metastasis, and lymphovascular invasion factors were relatively small, which may in turn cause strong error and bias in the survival analysis.

Recent advances in the role of tumor-associated neutrophils reveal that in the tumor microenvironment, neutrophils have varied functions and have been classified using different terms, including N1/N2 neutrophils, tumor-associated neutrophils (TANs), and polymorphonuclear neutrophil myeloid–derived suppressor cells (PMN-MDSCs) (11, 39). Fridlender et al. first delineated antitumorigenic and protumorigenic neutrophils, termed N1 and N2, respectively (40). Antitumor neutrophils can directly kill tumor cells through release of reactive oxygen species (ROS) and reactive nitrogen species (RNS). In contrast, protumor neutrophils can release matrix metalloproteinase 9 (MMP9), which promotes angiogenesis and dissemination of tumor cells, and they can also suppress NK cell function. PMN-MDSCs, as well as other protumor neutrophils, can suppress CD8 T-cell function (11, 39, 40). In our work, tumor-infiltrating neutrophils that were subsequently internalized by tumor cells played a protumorigenic role as N2 type, which better explains our conclusion: the FNiT as a novel predictor is significantly negatively associated with RFS and DSS of patients with BMSCC.

Of course, we still have large amounts of work to do, and our ultimate goal is to provide novel targets and strategies for the diagnosis, treatment and prognosis management of BMSCC, and to create a new field for the fundamental research on the tumor microenvironment of BMSCC.

### Limitations

The limitations of the study should be acknowledged. First, our current study has a retrospective design, so the inherent bias might reduce the statistical power. Second, the clinicopathological factors were disproportionally distributed in the enrolled patients, which resulted in obvious error in the statistical analysis.

## Conclusions

The FNiT as a novel predictor is significantly negatively associated with both the RFS and DSS of patients with BMSCC.

## Data Availability Statement

All datasets generated for this study are included in the article/supplementary material.

## Ethics Statement

The studies involving human participants were reviewed and approved by: The Zhengzhou University Institutional Research and Ethics Committee. The patients/participants provided their written informed consent to participate in this study. Written informed consent was obtained from the individual(s) for the publication of any potentially identifiable images or data included in this article.

## Author Contributions

JF wrote the manuscript. QS, SL, WD, and QF made manuscript preparation suggestions and final manuscript revision. QS funded the research. YY and MC did routine work on approval procedures of IRC. JF and MZ collected clinical data and performed statistical analysis. JQ and RL performed fluorescence staining assay and figure preparation. All authors contributed to the article and approved the submitted version.

## Funding

This research was supported by the National Natural Science Foundation of China (8157110152, 81872314).

## Conflict of Interest

The authors declare that the research was conducted in the absence of any commercial or financial relationships that could be construed as a potential conflict of interest.

## Abbreviations

FNiT, Frequency of heterotypic neutrophil-in-tumor structure; BMSCC, Buccal mucosa squamous cell carcinoma; HNSCC, Head and neck squamous cell carcinoma.
